# Teledentistry during COVID-19 pandemic: scientometric and content analysis approach

**DOI:** 10.1186/s12913-022-08488-z

**Published:** 2022-09-01

**Authors:** Abdullah Mahdavi, Rasha Atlasi, Roya Naemi

**Affiliations:** 1 Department of Health Information Management, School of Paramedical Sciences, Ardabil University of Medical Sciences, Ardabil, Iran; 2 Dental Research Institute, Tehran University of Medical Sciences, Tehran, Iran; 3 Endocrinology and Metabolism Research Center, Endocrinology and Metabolism Clinical Sciences Institute, Tehran University of Medical Sciences, Tehran, Iran

**Keywords:** Teledentistry, Covid-19, Telemedicine

## Abstract

**Introduction:**

During Covid-19 pandemic, people have been reluctant to visit dentist due to the fear of infection. Dentists have also suffered from severe turmoil. Teledentistry has been considered as a key strategy for managing people’s oral health. The aim of this study was to investigate teledentistry services in the world during the Covid-19 pandemic and to learn from different experiences.

**Materials and methods:**

The Web of Science database was searched by scientometric analysis approach, using keywords such as Covid-19, telemedicine, teledentistry and synonymous keywords derived from MeSH. Consequently, 94 articles were extracted from which, 15 articles related to teledentistry during Covid-19 were included in the study, considering the inclusion and exclusion criteria.

**Results:**

Scientometric analysis and illustration of retrieved articles were performed to identify authors, organizations and countries, and to review cooperation and collaboration networks in this field. Teledentistry was studied during Covid-19 in countries such as China, United States, Japan, Italy and United Kingdom. Types of the software used for communication between dentists and patients were WeChat, BigWord telephone translator, iGAM, WhatsApp, Attend Anywhere (AA), DeRS. The software used for data storage were Mouthwatch TeleDent, and Proforma. For data processing and decision making decision tree algorithms, automated algorithms were employed.

**Conclusion:**

Teledentistry has the potential to improve the provision of dental services. A fundamental review and careful planning is required to provide such services, particularly in developing countries. Furthermore teledentistry needs more in-depth studies in order to overcome existing and future obstacles and risks while taking advantage of its benefits.

## Introduction

Covid-19 has posed significant challenges for dentists and patients, because direct patient-dentist contact and close proximity to patient’s oropharynx increase the risk of virus infection [1]. During Covid-19 pandemic, people have been reluctant to visit dentists due to the fear of infection, and front-line health care workers such as dentists have suffered from severe turmoil, anxiety, poor sleep quality, and increased risk of mental illness [2]. Regulatory agencies have recommended social distancing and home quarantine to prevent close contact between individuals and reduce the spread of infection [3]. The Italian scientific dental association has provided recommendations for the management and triage of patients before and after dental treatment, and acknowledged that by suspending dental care, the number of patients in need of hospitalization may increase in the future, so dentists can only provide emergency services and in other cases, they can treat patients digitally using tools such as telemedicine to ensure patient safety, minimize frequent contact with patient and cut the Covid-19 transmission chain [4].

Cook, for the first time in 1997, described teledentistry as the use of videoconferencing in remote diagnosis and counseling [5]. Teledentistry, also called e-dentistry [6], now encompasses the use of electronic patient records, video, and 3D digital images for diagnosis and consultation and is not limited to videoconferencing [7]. Teledentistry has, therefore, been considered as a key strategy for managing people’s health while maintaining a safe distance and limiting close contact with them. Teledentistry includes remote diagnosis, remote monitoring, remote treatment, and remote rehabilitation through virtual platform technology [3]. Telemedicine services, including teledentistry, have grown globally as public awareness has grown with a forecast of 20% further growth over the next 5 years, and revenue growth from $38 billion in 2018 to about $130 billion by 2025 in USA [8]. In a study by Vanka and colleagues, the reasons for using technology in dental services included: reducing the risk of Covid-19, communicating properly with a dentist during the pandemic, having access to dental care more quickly, and saving patient’s time and money [1].

According to Singer and colleagues, emergency dental problems and surgeries cannot be controlled through telemedicine and during the pandemic, structural changes in dental offices and clinics are necessary to reduce virus transmission between dentists and patients [4]. In Crawford and Taylor’s study of barriers to remote dental practice prior to Covid-19 epidemic, factors such as forensic concerns, dentists’ lack of confidence in using computers and online systems, software and training processes, crowded clinics, satisfaction with current system, costs of installing necessary systems and technologies, dentists’ referral fees, impossibility to perform practical interventions remotely, patient safety and data protection were among the main concerns of dentists [6].

Consequently, Covid-19 pandemic has been one of the biggest challenges of health care systems and has forced health care organizations to rapidly change their patient care approaches [9]. Therefore, the present study aimed to investigate the teledentistry services offered by dentists around the world during Covid-19 and to learn from their experiences through scientometric analysis approach. Scientometric approach utilizes quantitative analysis methods and software programs [10] to evaluate the scientific publications of researchers and scientific organizations. It also evaluates topics, journals or countries to compare and rank them for showing an overview of scientific performances and helps policy makers [11, 12].

## Materials and methods

**Fig. 1:**
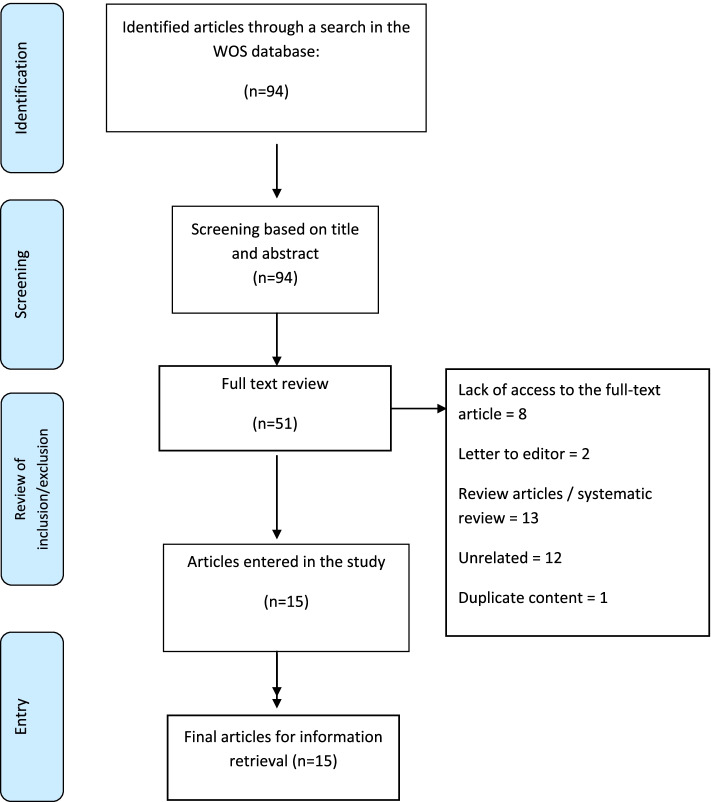
The screening process of articles included in the study

In this study, a structured and comprehensive search was conducted in the international electronic database of Web of Sciences (WOS), using keywords such as Covid-19, Telemedicine, Teledentistry and other synonymous words extracted from the MeSH. The search strategy and number of results are presented in Table 1. In total, 94 articles were extracted from WOS database that had been published as of January 1st, 2020 to January 25th, 2022 in English language. First, the title and abstract of the articles were reviewed based on inclusion criteria;, namely, all types of studies conducted on teledentistry during Covid-19 pandemic as clinical trial, cohort and prospective studies. Systematic review articles, reviews and letters to editor were excluded from the study. After screening the title and abstract of the articles, 51 articles were selected for the full text review based on the inclusion and exclusion criteria, and finally 15 articles were included in the study. The reasons for removing articles are listed in Fig. 1. Finally, after retrieving the articles, the scientometric analysis of selected articles was carried out in terms of the most producing country, the cooperation network of these countries, the number of citations of articles in different countries, partner institutions in the production of these articles, co-authors network, journals along with the number of articles and citations, impact factor and quarter, the co-occurrence use of all keywords in the 15 articles included in the present study, and the relationship between authors, institutions and countries.

**Table 1:** Search Strategy used to conduct a search in the WOS Database

Search strategy	Number of result
TS = ((COVID-19 OR COVID19 OR SARS-CoV-2 OR coronavirus* OR Deltacoronavirus* OR Alphacoronavirus OR Betacoronavirus OR Gammacoronavirus OR Deltacoronavirus OR (corona AND Virus*) OR 2019-nCoV OR SARS2 OR “SARS 2”) AND (Telemedicine OR (Mobile AND Health) OR mHealth OR m-Health OR Telehealth OR eHealth OR e-Health OR e-medicine OR e-care OR ((Video OR Remote) AND Consultation*) OR Telecommunication* OR (TELE NEAR/3 (medicine OR health* OR care* OR Communication* OR DENT*))) AND (Tooth OR Teeth OR DENT*))	94

Then, the results of scientometric analysis were presented in the form of statistical tables (number, frequency, percentage) and data visualization. Then, the contents of 15 articles related to the purpose of this study were analyzed and the information were divided into 8 columns containing title of included articles, citations of each article, centers providing teledentistry services, teledentistry services, software/ app used, advantages of teledentistry, disadvantages of teledentistry and barriers/risks of teledentistry.

## Findings

### Scientometric analysis

**Fig. 2:**
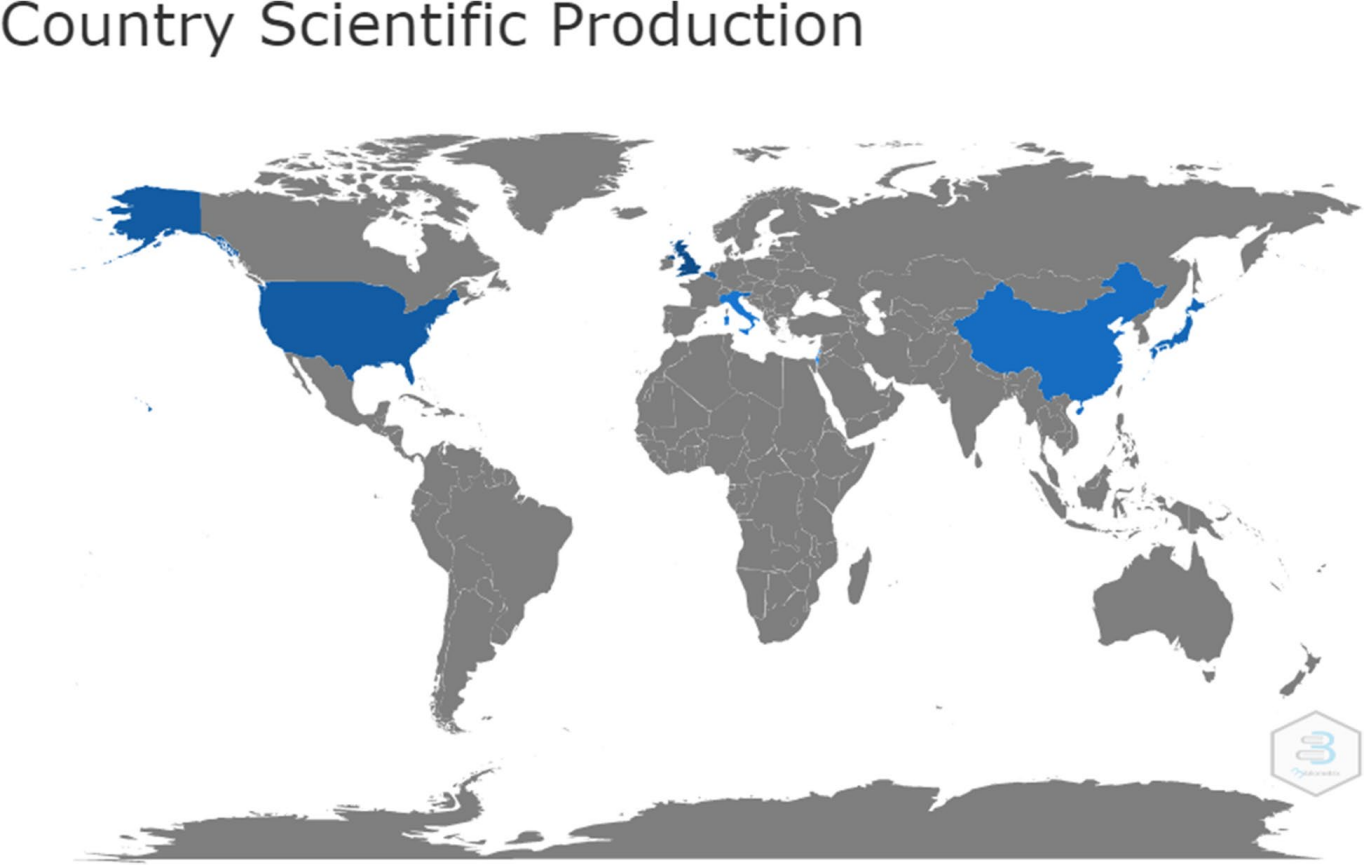
Number of article productions by country related to teledentistry during Covid-19 pandemic found in the WOS database

In the first part of the findings, we present the results of scientometric analysis of 15 articles related to teledentistry during Covid-19 pandemic. Most of these articles (8 articles) have been published in 2021 (53.333%), and the rest (6 articles, 40%) in 2022 and 2021 (1 article). These articles have been conducted in collaboration with different countries, which can be seen in the figure below (Fig. 2). The largest producer of articles in this field was the UK with 12 articles, followed by USA (8 articles), Belgium, Japan and Slovenia (6 articles).

**Fig. 3:**
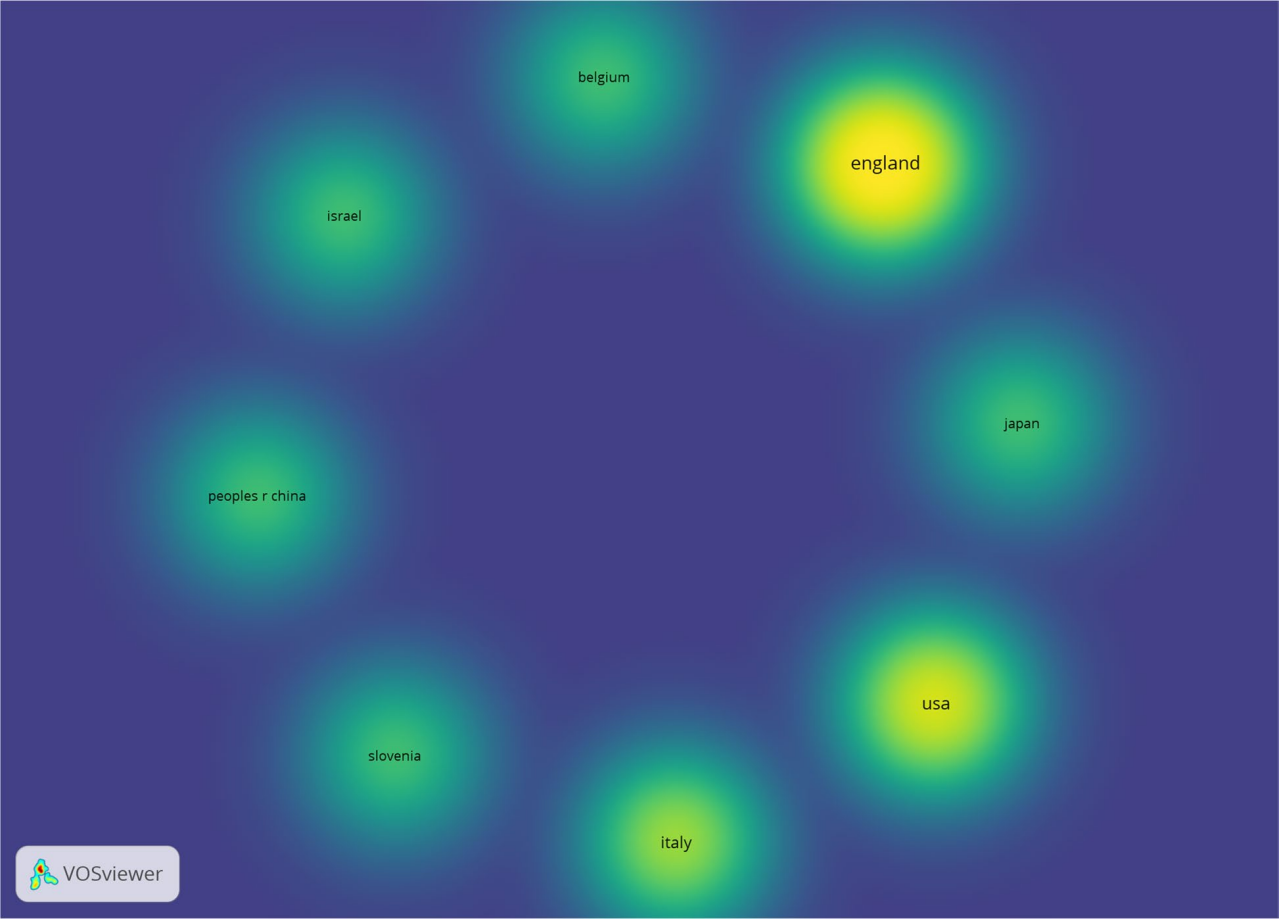
The cooperation network of countries producing articles related to teledentistry during Covid-19 pandemic found in the WOS database

The cooperation network of these countries is in the form of 8 separate and unconnected clusters. Britain, United States and Italy have been more active than other countries in producing articles (Fig. 3).

**Fig. 4:**
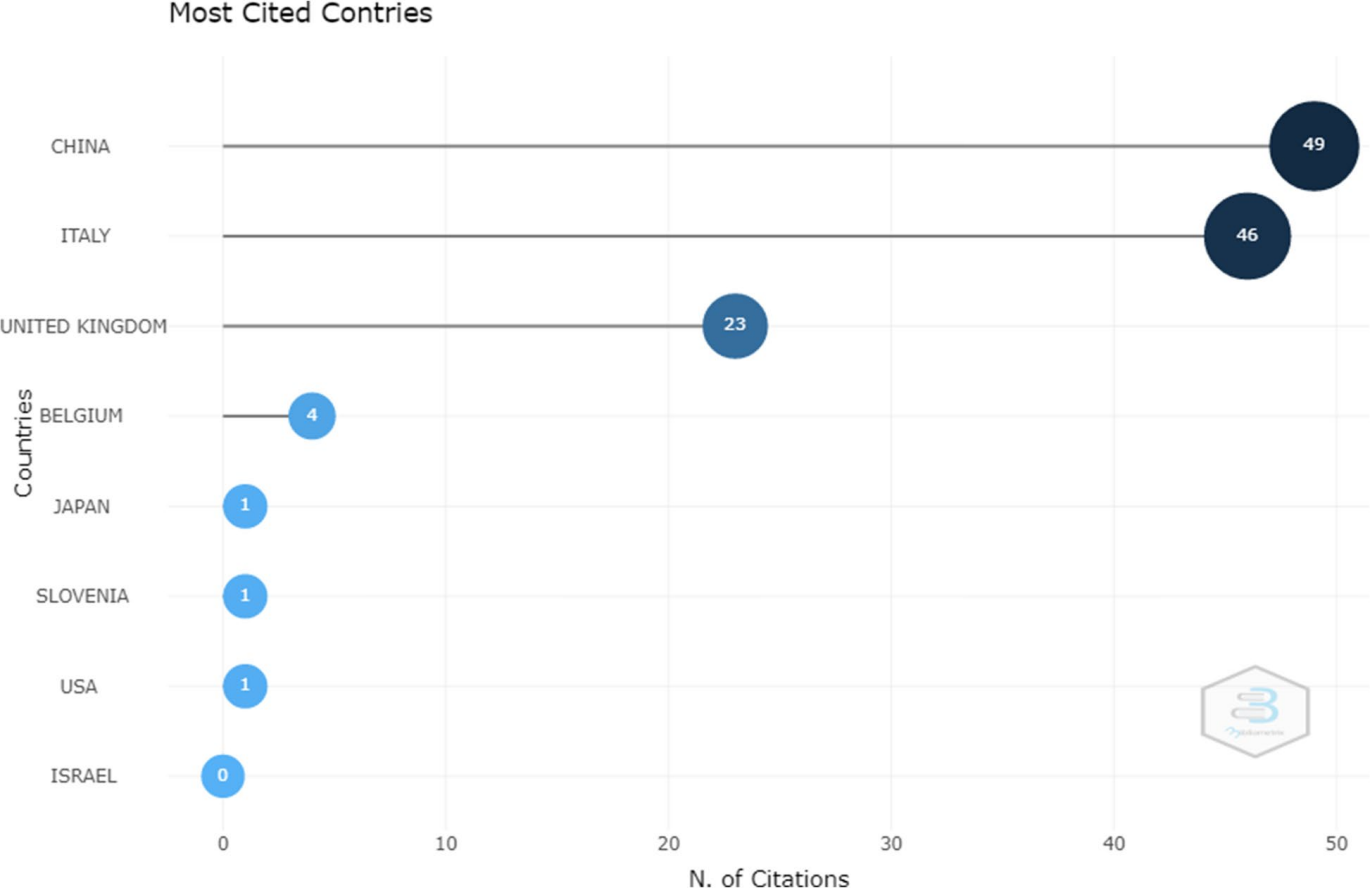
Number of citations to articles in different countries found in the WOS database

An examination of the number of citations to articles in different countries also showed that China was in the first place with 49 citations, followed by Italy with 46 citations and United Kingdom with 23 citations in the second and third places (Fig. 4).

**Fig. 5:**
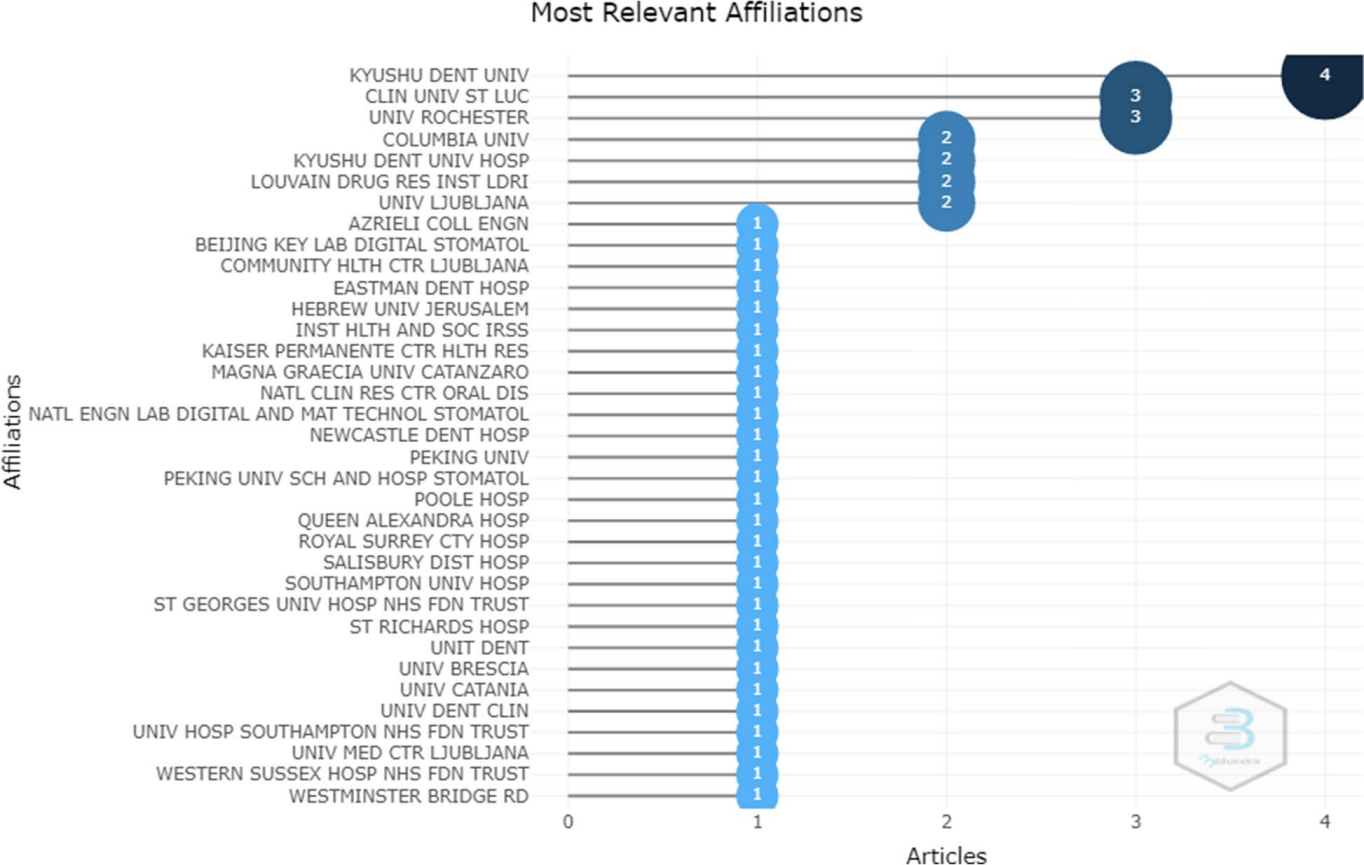
Partner institutions that helped to produce articles related to teledentistry during Covid-19 pandemic found in the WOS database

The results of analysis of partner institutions that helped to produce these articles also showed that Kyushu Dent University with 4 articles and then, Cliniques universities Saint-Luc and University of Rochester with 3 articles each were in the top spots (Fig. 5).

**Fig. 6:**
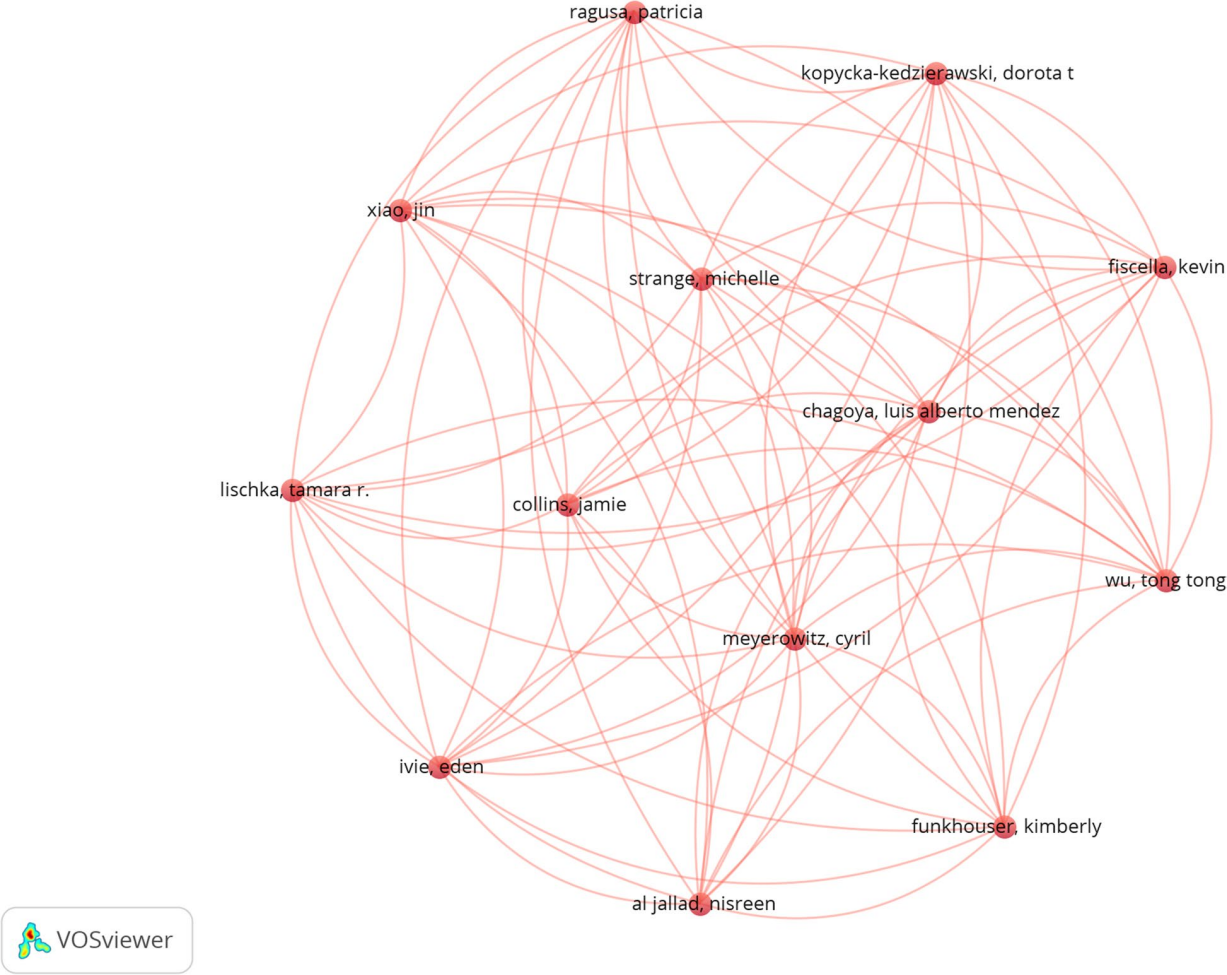
Authors’ collaboration network

The co-authored network of these authors was as follows: The largest co-authored network consisted of 13 authors in a cluster with 78 connections (Fig. 6).

**Fig. 7:**
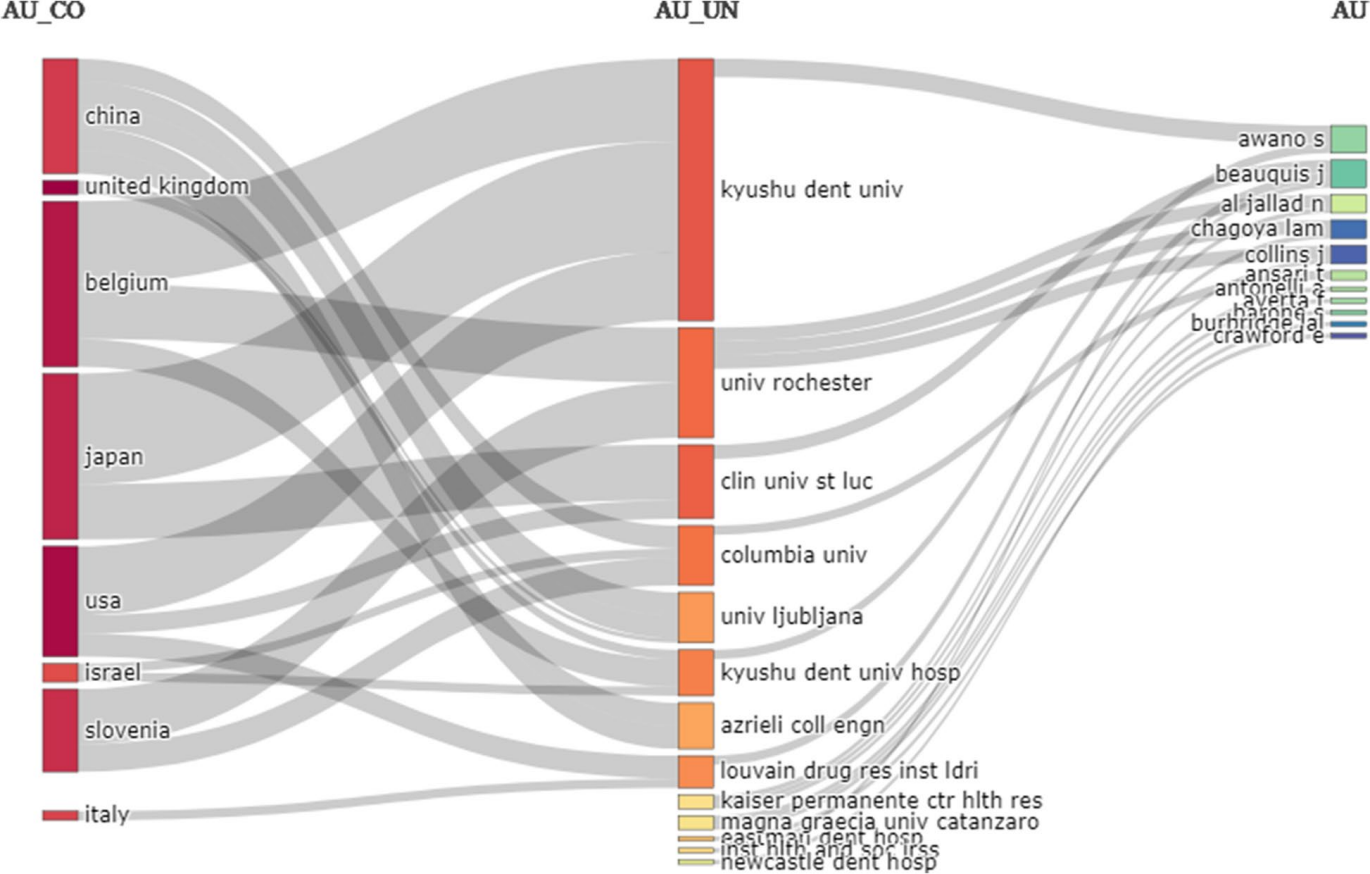
Relationship between authors, institutes and countries producing articles related to teledentistry during Covid-19 pandemic found in the WOS database

In general, the relationship between authors, institutions and countries (top 20) is presented in Fig. 7.

With regard to journals that published these articles, table below shows the journals along with the number of articles and citations as well as their impact factor and quarter (Table 2).

**Table 2:** Number of articles, citations, impact factor and quarters of journals that published articles used in this study

Source	IF	Q	Documents	Citations
British Dental Journal	1.626	Q4	1	1
British Journal Of Oral & Maxillofacial Surgery	1.651	Q4	1	23
Clinical And Experimental Dental Research	–	–	1	1
Clinical Oral Investigations	3.573	Q1	1	49
Community Dental Health	1.349	Q4	1	0
European Archives Of Pediatric Dentistry	–	–	1	2
International Journal Of Environmental Research And Public Health	3.39	Q1	1	46
Jmir Mhealth And Uhealth	4.773	Q1	1	0
Jmir Research Protocols	–	–	1	0
Journal Of Dental Research	6.116	Q1	1	4
Journal Of Dental Sciences	2.08	Q3	1	1
Journal Of Orthodontics	–	–	1	5
Journal Of Public Health Dentistry	1.821	Q3	1	1
Journal Of The American Medical Informatics Association	4.497	Q1	1	0
Open Dentistry Journal	–	–	1	3

**Fig. 8:**
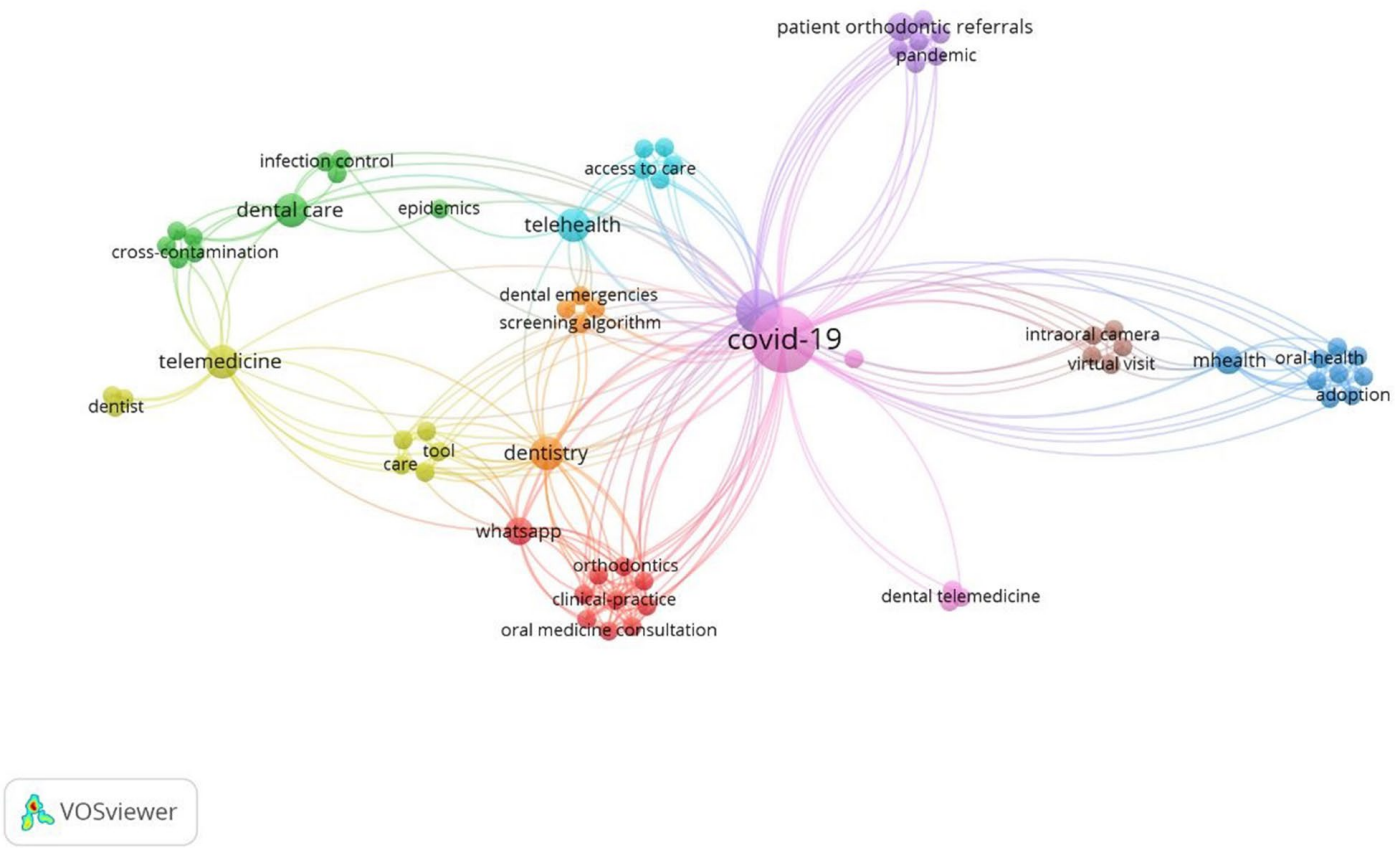
The co-occurrence of all keywords in articles related to teledentistry during Covid-19 pandemic found in the WOS database

In this study, the co-occurrence of all keywords in the articles was also analyzed. The results showed that 70 keywords were assigned for these 15 articles. Among these keywords, 67 keywords were connected to each other and made a network with 317 links among them (Fig. 8).

**Fig. 9:**
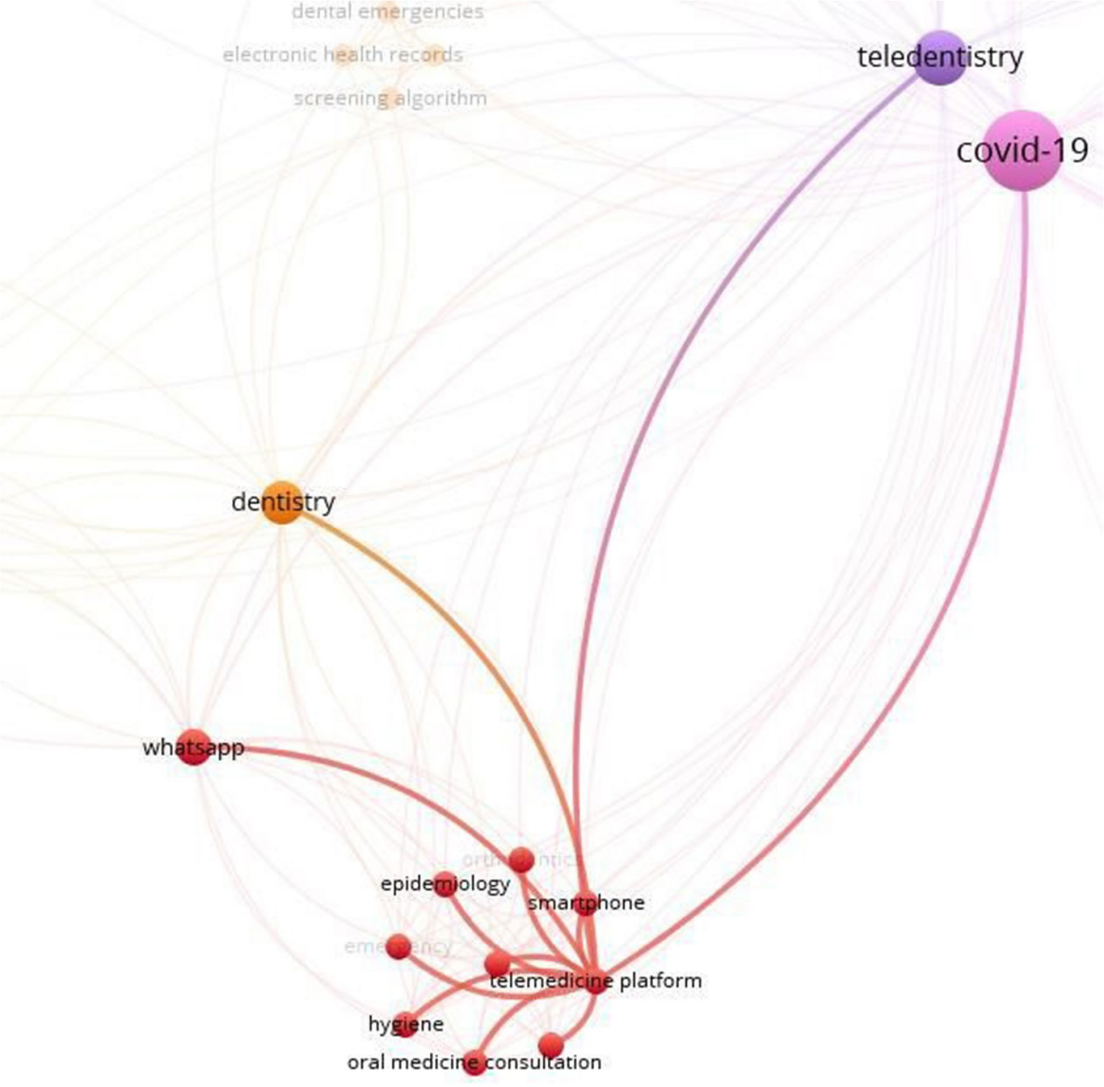
One cluster selected from the network of keywords in articles related to teledentistry during Covid-19 pandemic found in the WOS database

Also these keywords formed 9 clusters. As can be seen, cluster 1 and its keywords are connected to each other and form part of the big network (Fig. 9).

### Content analysis of included articles

The second part of the findings, as shown in table 3, presents the health centers providing teledentistry services during Covid-19 which included the General Dental Hospital in China, US National Dental Practice-Based Research Network, Newcastle Dental Hospital, The Hebrew University-Hadassah School of Dental Medicine, Green Leaf Dental Care at St. Louis Metropolitan Area, Columbia University’s College of Dentistry, Pediatric Dentistry of St. Thomas Hospital, Slovenia, Japan Dental Clinic, Oral and Periodontal Surgery and Oral Pathology, Department of Oral Surgery and Pathology at University of Magna Graecia Catanzaro in Italy, UK Orthodontic Hospital, Oral and Maxillofacial Units located at five NHS Hospitals, St. Luke University Clinic (Brussels, Belgium), NHS Foundation Hospitals in South West London, and Private Dental Clinics in Italy.

**Table 3:** Summery of the findings of 15 studies entered in the present study

The title of included articles	Citations	Centers providing teledentistry services	Teledentistry services	Software/ app used	Advantages of teledentistry	Disadvantages of teledentistry	Barriers/risks of teledentistry
Health services provision of 48 public tertiary dental hospitals during the COVID-19 epidemic in China [13]	49	China General Dental Hospital	Tele-consultation Remote assessment for identify the population at risk	Access way: web-based mobile WeChat Data storage: - Data processing:-	Clinical data integration, access to patients’ medical records, ease of referring patients to hospitals or dental clinics, improving medical care in rural and remote areas	–	–
Assessment of an Innovative Mobile Dentistry eHygiene Model Amid the COVID-19 Pandemic in the National Dental Practice-Based Research Network: Protocol for Design, Implementation, and Usability Testing [14]	0	US National Dental Research Network (pilot Study)	Tele-examinations Electronic dental hygiene Oral disease screening	Access way: - Data storage: Mouthwatch TeleDent Data storage: - Data processing:-	Enhancing immunity during COVID-19 and other emerging epidemics, reducing the consumption of personal protective equipment (PPE)	–	–
Role of teledentistry in paediatric dentistry [15]	1	Newcastle Dental Hospital	Tele-consultation Tele-triage	Access way: BigWord Translator for patients who needed English translation services, Data storage: Microsoft Excel Data processing:-	Reducing unnecessary travel of patients, reduction of unnecessary face-to-face examination, reduce the waiting list, increasing immunity in the era of Covid-19, reducing anxiety in patients with dental or social phobia, access to dental care for non-English speakers.	Not answering calls due to using withheld numbers, the quality of teleconsultation was dependent on the quality of radiographics, distractions during phone calls and interference with receiving messages	The risk of misdiagnosis and mismanagement of patients due to the quality of received images, patient cooperation and the quality of dentist and patient IT devices, financial implications and disclosures of data/ Teledentistry services unsuitable for certain groups of patients, such as the hearing impaired or those requiring additional assistive communication devices, lack of access to teledentistry services due to not having a mobile phone or computer
Using an mHealth App (iGAM) to Reduce Gingivitis Remotely (Part 2): Prospective Observational Study [16]	0	The Hebrew University-Hadassah School of Dental Medicine	Remote Monitoring patients to reduce gingivitis Remote oral health education	Access way: iGAM M Health app Data storage: - Data processing:-	Promote gum health	–	–
Teledentistry applications for mitigating risk and balancing the clinical schedule [8]	1	Green Leaf Dental Care at St. Louis Metropolitan Area	Tele-triage Tele-consultation Hygiene assessment Patient follow-up	Access way: synchronous or asynchronous videoconference	Reducing contact between people during the Covid-19, reducing the volume of patients, reducing waiting time, eliminate the use of PPE	Lack of key functions for easy and reliable workflow management in current software platforms, lack of communication in current software platforms Need more clinical data such as radiographs for differential diagnosis	–
Rapid deployment of an algorithm to triage dental emergencies during COVID-19 pandemic [17]	0	Columbia University College of Dentistry	Tele-visit Tele-triage for Covid-19 symptoms and pain score	Access way: - Data storage: - Data processing: Decision tree algorithm for determining the course of treatment by following the instructions of CDC^a^, ADA^b^ and CDM^c^	Optimal access to emergency dental services during Covid-19 Accurate screening of patients who needed emergency care based on a decision-making algorithm	–	–
Paediatric dental A&E service during the COVID-19 pandemic in the Greater London area [18]	2	Pediatric Dentistry of St. Thomas Hospital	Tele-triage using the guidance of the Royal College of Surgeons of England and the Scottish Dental Clinic Effectiveness Program	Access way: secure email to send photos Data storage: Microsoft Excel spreadsheet Data processing	Effective and safety	The three-way call system was not available in urgent cases to solve the problem of patients who do not know English. Phone counseling with parents and not children may affect the reliability of complaints.	–
Urgent dental care on a national level during the COVID-19 epidemic [19]	1	Slovenia at national level	Tele-triage	Access way: Telephone Data storage: - Data processing:-	Effective and safe dental care in the era of COVID-19 Reducing consumption of PPE and its efficient distribution among care providers	Lack of standardization in the data collection phase	Financial implications, involvement of a large number of dental health care workers in data collection and potential impact on data consistency
Effect of COVID-19 on dental telemedicine in Japan [20]	1	Japan Dental Clinic, Oral and Periodontal Surgery and Oral Pathology	The first examination face-to-face and follow-up by phone or online or video	Access way: Telephone, videoconference Data storage: - Data processing: -	Prevention of the spread of COVID-19 in young people and people with high IT literacy	–	Lack of IT literacy of the elderly to use smartphones and tablets Low reimbursement of teledentistry compared to face-to-face examination, challenge in accurate diagnosis due to lack of access to visual information, medical history, systemic diseases of the patient via teleconsultation
Can Teledentistry Improve the Monitoring of Patients during the Covid-19 Dissemination? A Descriptive Pilot Study [21]	46	Department of Oral Surgery and Pathology at University of Magna Graecia Catanzaro, Italy	Tele-consultation of patients with chronic diseases Remote examination of patients’ first visit	Access way: WhatsApp Data storage: hard disks in compliance with the GDPR^d^ Data processing: -	Reduce cost and waiting time Establishing a strong relationship between the doctor and the patient in the recovery process	Cost security Data privacy and its implications	
The effective use of an e-dentistry service during the COVID-19 crisis [6]	5	UK Orthodontic Hospital	Tele-consultation Tele-monitoring of orthodontic services	Access way: AA software, DeRS, phone 141, NHS Email Data storage: - Data processing: -	Saving time and money especially for people in remote areas, reducing waiting time, reducing environmental impact	Limitation in active treatment and adjustment of orthodontic equipment of patients, need to interact with patients in orthodontic services	Need to interact with orthodentis
Provision of emergency maxillofacial service during the COVID-19 pandemic: a collaborative five centre UK study [9]	23	Multicenter Study including Oral and Maxillofacial Units based at five NHS Hospitals	Tele-consultation	Access way: web-based application, telephone, video call Data storage: web-based application Data processing: automated algorithms	Reduction in the number of patients The willingness of patients to teleconsultation due to saving money and time even after the pandemic	Different access levels of some treatment centers to video equipment or strong internet connections	Different levels IT equipment in treatment centers
Dental Emergencies Management in COVID-19 Pandemic Peak: A Cohort Study [22]	4	St. Luke University Clinic (Brussels, Belgium)	Tele-triage Remote management of patients Tele-consultation	Access way: Telephone, Email, Data storage: systematic form Data processing:-	Reduction of patient admissions, success in emergency management of patients and postponing definitive treatment to a month later	–	Sending images electronically
Dental Public Health in Action: Utilising a telephone triage system to run an Urgent Dental Care Hub during the COVID-19 pandemic [23]	0	NHS Foundation Hospitals in South West London	Tele-triage Tele-consultation	Access way: Call 111, email Data storage: Proforma Data processing:-	Facilitate emergency dental care	Inability to perform clinical examination and radiographic observation of the patient through telephone triage	–
COVID-19 and Orthodontics: An Approach for Monitoring Patients at Home [24]	3	Private Dental Clinics in Italy	Tele-monitoring Remote management of orthodontic patients	Access way: WhatsApp Data storage: - Data processing:-	High Patient cooperation, high speed response due to the use of WhatsApp	The quality of the images was different depending on the smartphone, which led to the orthodontist requesting retakes	Different quality of the images

*CDC* Center for Disease Control and Prevention, *ADA* American Dental Association, *CDM* The College of Dental Medicine, *GDPR* General Data Protection Regulation

Remote dental services provided by the above mentioned centers included specialized telephone or video consultation, tele-triage, remote examination, screening of patients for Covid-19 symptoms, patient education and monitoring, electronic dental hygiene, and remote follow-up of treatment and management. The infrastructure used to deliver teledentistry services included internet, telephone, smartphone with high camera resolution, video call, SMS, email, central database, electronic prescribing, electronic referral system, electronic patient record, patient management system and PACS.

Concerning the software used in teledentistry, it was divided into three categories: software used for doctor-patient communication (access way), data storage software, and data processing software. Types of the software used for communication between dentists and patients were WeChat, BigWord telephone translator, iGAM, WhatsApp, Attend Anywhere (AA), DeRS^1^. The software used for data storage were Mouthwatch TeleDent, and Proforma. For data processing and decision making decision tree algorithms, automated algorithms and systematic forms were employed (Table 3).

## Discussion

The scientometric analysis of articles used in this study, which were related to teledentistry during Covid-19 pandemic, showed factors such as the countries producing most articles in this field, the cooperation network of these countries, the number of citations of articles in different countries, partner institutions that helped to produce these articles, the co-authorship network, the journals along with the number of articles and citations, the impact factor and quarter of articles, the co-occurrence of all keywords in the articles and the keywords assigned by the WOS database, as well as the relationship between authors, institutions and countries. The results of this study indicated that several efforts have been made by different countries to study teledentistry during Covid-19 pandemic.

Most of the articles were conducted in England and most citations belong to the articles from China. Furthermore, “Journal of Dental Sciences” with the highest impact factor (6.116) had only 4 citations and two journals, with IF around 3, had the most citations (more than 40 citations). This indicates that impact factor might not be an important element to cite a journal’s articles. The number of included articles in short timespan was not high. So all of the authors in these articles participated and collaborated in one article so we can find out the more active authors in the field.

In addition to scientometric analysis of included articles for realizing the overview of the production in the field, the content of these articles was also analyzed. Teledentistry has been made possible through Information Technology (IT), internet, patient’s online electronic record, digital devices such intraoral cameras, webcams, and remote computer monitors [25]. Remote assessment of dentists should be done when they feel that they can complete a treatment after performing adequate evaluation. Remote prescribing should be clinically justified [15]. Using technologies such as interactive live examination, video and specialized chartrooms between dentists, distance patient education, tele-triage, tele-monitoring and treatment of oral lesions, tele-consultation between dentists, etc. reduce the risk of Covid-19 infection [25]. Sharma and colleagues have divided the applications of pediatric teledentistry into three general categories of education and promotion of oral health, remote diagnosis and monitoring, and behavioral guidance [25].

In addition to reducing the cost of travel, teledentistry can reduce the anxiety of people with dental phobia, prioritize patients, reject inappropriate referrals without face-to-face consultation, increase the range of services in the field of oral health, and give parents the opportunity to choose pre-appointment treatment options [4, 6, 15, 22]. Also, during Covid-19 pandemic, teledentistry can be used as a suitable alternative for patients who do not need immediate face-to-face examination [8, 15]. It also prevents the accumulation of patients in the office, which reduces the spread of infection [8, 14, 19, 26].

Giudice and colleagues, used WhatsApp for remote consultation and follow-up of patients. They also stated that, remote consultation and constant monitoring of patient improve patient’s participation and adaptation to treatment, as well as physician-patient relationship [21, 24]. Prior to Covid-19 pandemic, teledentistry services may have been difficult to provide but it has become easier for dentists and patients to accept such services [8]. Subhan and colleagues in their study stated that dental specialists in Pakistan before Covid-19 pandemic did not have enough knowledge about teledentistry but now their knowledge about the advantages of teledentistry and its implementation has been significantly increased [27].

In a qualitative study conducted by Plessas and colleagues during Covid-19 pandemic, the dentists stated that they felt good about helping patients, reducing their pain and suffering, the professional satisfaction of doing the right thing. Also, factors such as strategic team working, collective coping strategies, effective organizational care, the ability to help, and the sense of pride have created a positive experience in them [2]. In contrast, feelings of frustration due to scattered communication, feelings of injustice in communication, high demand of patients, complex decisions, uncertainty about safety, inefficient communication channels, lack of commitment to remote video consulting, the quality of referrals, communication with other health care providers, slow communication, poor support, high workload without extra payment, change in prescribing behavior, irreversible decisions, out-of-date computer systems, perceptions of patient ability in interaction, and lack of radiography have been mentioned as the negative points of teledentistry [2, 8, 9, 15, 23, 24].

However, the main concern with teledentistry might be attributed to the misdiagnosis and mismanagement of patient due to the following factors: the quality of received images and radiographies, sense and degree of patient cooperation, the quality of IT devices belonging to dentists and patients, lack of access to medical history, systemic diseases of the patient which may harm the patient, and medical and legal issues [15, 20, 26]. Accuracy of diagnosis depends on using the same and professional standards, the quality of radiographic images, quality of video calls, microphone resolution, good quality internet patient cooperation and quality of electronic devices [6, 7, 9, 15, 17, 19, 24]. These factors are influenced by the socio-economic status of patient and the level of patient’s knowledge about IT [7]. Al-Shaya and colleagues in their study acknowledged that teledentistry with the mobile phone photo is not as accurate as clinical examination through radiography but the initial diagnosis of caries in children with teledentistry has acceptable reliability [28].

Clinical examination and remote screening of patients, for example through WhatsApp images, can be a suitable, reliable and cost-effective tool in a low-resource environment [22, 29, 30]. Barriers to teledentistry include patients’ lack of knowledge about IT, poor and inadequate IT literacy of dentists, unfamiliarity of dentists and patients with technology, limitations in infrastructure (such as poor internet access, lack of hardware, cost and efficiency of equipment), low video quality, audio-video mismatch that can be replaced by direct communication (simultaneous communication), how to store, send and share patient information, confidentiality and protection of patient information, organizational changes in accepting teledentistry, non-acceptance of teledentistry services by relevant authorities including insurance companies, lack of transparency in reimbursement, non-cooperation of other medical centers, inadequate circulars, cost of installing teledentistry equipment and resistance of dentists to new technologies [3, 9, 19, 20, 22, 24, 25, 31].

The field of dentistry also needs a fundamental review and rapid and planned changes to provide teledentistry services to vulnerable populations such as children, elderly, people with special needs and people with low socio-economic status. Therefore, this development requires systematic planning, analysis and feasibility studies, so further studies should be conducted to improve its efficiency [32]. As a consequence, educating dentists about the use of technology, obtaining informed consent from patients before starting any treatment, conducting further research on the improving the effectiveness of teledentistry in different dental clinics, examining the cost-effectiveness of procedures, assessment of patient satisfaction, long-term socioeconomic, psychological, and physical effects of teledentistry services, development of, reliable and effective software for patient management, optimization of software and procedures and considering taxes, obtaining licenses, new business models, developing guidelines and clear rules for reimbursement of services, violations and forensics are among the solutions to overcome these challenges [6, 9, 13, 18, 20, 21, 23, 25].

Considering that teledentistry is more compatible with providing innovative solutions to problems, while limiting the exposure of dentists and patients to Covid-19 [8], further studies are needed to probe into the similarities and differences of teledentistry services in different geographical areas, particularly in developing countries in order to take strategic steps towards addressing the existing and forthcoming challenges while taking advantage of teledentistry [15] because, as shown in Figs. 2 and 3, a few of developing countries collaborated to produce documents in the field of teledentistry during COVID-19. However, this topic seems to be important and useful in all countries including developing countries.

With the outbreak of Covid-19, health care organizations have faced a sudden increase in demand for the use of IT in health care. Teledentistry, not only during Covid-19 pandemic, but also under normal circumstances can be a useful and complementary tool for face-to-face assessment of patients before treatment, patient education and monitoring, preventive care, post-treatment follow-up, diagnosis of diseases and treatment planning, which reduce anxiety in patients and lead to improved outcomes of patient management. Achieving this goal requires the cooperation of Ministry of Health and Medical Education, insurance organizations, heads of hospitals and clinics and other relevant authorities. Some of the strengths of this study include the review of articles in the most authoritative database, and scientometric analysis of selected articles. In regard to the weaknesses of this study, we can refer to the lack of review of other databases.

## Conclusion

The results of present study showed the numerous efforts have been made by different countries to study teledentistry during Covid-19 pandemic. Teledentistry through IT has facilitated live and interactive examination of patients, videoconferencing, distance education of patient, tele-triage, tele-monitoring of treatment process, and tele-consultation, which have ultimately increased client satisfaction. Teledentistry, in addition to reducing the cost of travel, reduces the anxiety of people with dental phobias by increasing the range of services in the field of oral health care. During Covid-19 epidemic, teledentistry has been a viable alternative for patients who do not need immediate and face-to-face oral assessment. By the use of teledentistry during the pandemic, dentists will able to provide dental care and reduce the chain of infection. In contrast, uncertainty about safety, inefficient communication channels, lack of commitment to video conferencing, and barriers to large-scale implementation of this technology have been mentioned as some of the barriers to teledentistry. The field of teledentistry requires a fundamental review, and rapid and planned changes to facilitate the provision of teledentistry services. Considering that teledentistry is effective in limiting the exposure of dentists and patients to Covid-19, more and in-depth studies are required in developing countries to address the existing and forthcoming challenges of teledentistry, while taking advantage of this new approach.

## Data Availability

The datasets used and/or analysed during the current study available from the corresponding author on reasonable request.

## Abbreviations

WOS: Web of Sciences
AA: Attend Anywhere
IT: Information Technology
DeRS: Dental Electronic Referral System

## Footnotes

1 Dental Electronic Referral System
